# 2-(4-Chloro­phen­yl)-2-oxoethyl 3-(trifluoro­meth­yl)benzoate

**DOI:** 10.1107/S1600536811020629

**Published:** 2011-06-11

**Authors:** Hoong-Kun Fun, Wan-Sin Loh, B. Garudachari, Arun M. Isloor, M. N. Satyanarayan

**Affiliations:** a X-ray Crystallography Unit, School of Physics, Universiti Sains Malaysia, 11800 USM, Penang, Malaysia; b Organic Chemistry Division, Department of Chemistry, National Institute of Technology – Karnataka, Surathkal, Mangalore 575 025, India; c Department of Physics, National Institute of Technology – Karnataka, Surathkal, Mangalore 575 025, India

## Abstract

In the title compound, C_16_H_10_ClF_3_O_3_, the two benzene rings are slightly twisted from each other, with a dihedral angle of 15.50 (8)° between the planes. In the crystal, inter­molecular C—H⋯O hydrogen bonds link the mol­ecules into a layer parallel to the *bc* plane.

## Related literature

For the background and applications of phenacyl benzoates, see: Sheehan & Umezaw (1973 ▶); Ruzicka *et al.* (2002 ▶); Litera *et al.* (2006 ▶); Rather & Reid (1919 ▶); Huang *et al.* (1996 ▶); Gandhi *et al.* (1995 ▶). For bond-length data, see: Allen *et al.* (1987 ▶). For the stability of the temperature controller used for the data collection, see: Cosier & Glazer (1986 ▶).
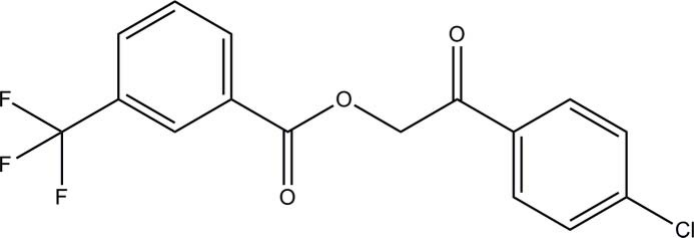


## Experimental

### 

#### Crystal data

C_16_H_10_ClF_3_O_3_
*M* *_r_* = 342.69
Monoclinic, 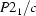

*a* = 14.3036 (7) Å
*b* = 12.1335 (6) Å
*c* = 8.5464 (4) Å
β = 101.444 (1)°
*V* = 1453.76 (12) Å^3^
*Z* = 4
Mo *K*α radiation
μ = 0.31 mm^−1^
*T* = 100 K
0.28 × 0.16 × 0.11 mm

#### Data collection

Bruker SMART APEXII CCD area-detector diffractometer
Absorption correction: multi-scan (*SADABS*; Bruker, 2009 ▶) *T*_min_ = 0.919, *T*_max_ = 0.966
15629 measured reflections
3822 independent reflections
2963 reflections with *I* > 2σ(*I*)
*R*_int_ = 0.046

#### Refinement

*R*[*F* ^2^ > 2σ(*F* ^2^)] = 0.048
*wR*(*F* ^2^) = 0.121
*S* = 1.04
3822 reflections
208 parameters
H-atom parameters constrained
Δρ_max_ = 0.52 e Å^−3^
Δρ_min_ = −0.33 e Å^−3^

### 

Data collection: *APEX2* (Bruker, 2009 ▶); cell refinement: *SAINT* (Bruker, 2009 ▶); data reduction: *SAINT*; program(s) used to solve structure: *SHELXTL* (Sheldrick, 2008 ▶); program(s) used to refine structure: *SHELXTL*; molecular graphics: *SHELXTL*; software used to prepare material for publication: *SHELXTL* and *PLATON* (Spek, 2009 ▶).

## Supplementary Material

Crystal structure: contains datablock(s) global, I. DOI: 10.1107/S1600536811020629/is2720sup1.cif

Structure factors: contains datablock(s) I. DOI: 10.1107/S1600536811020629/is2720Isup2.hkl

Supplementary material file. DOI: 10.1107/S1600536811020629/is2720Isup3.cml

Additional supplementary materials:  crystallographic information; 3D view; checkCIF report

## Figures and Tables

**Table 1:** Hydrogen-bond geometry (Å, °)

*D*—H⋯*A*	*D*—H	H⋯*A*	*D*⋯*A*	*D*—H⋯*A*
C1—H1*A*⋯O1^i^	0.95	2.53	3.205 (2)	128
C4—H4*A*⋯O3^ii^	0.95	2.53	3.289 (2)	137
C8—H8*A*⋯O3^iii^	0.99	2.48	3.474 (2)	177

Symmetry codes: (i) 
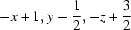
; (ii) 
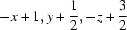
; (iii) 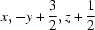.

**Fig. 1.**
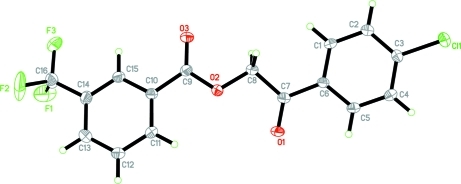
The molecular structure of the title compound, showing 50% probability displacement ellipsoids and the atom-numbering scheme.

**Fig. 2.**
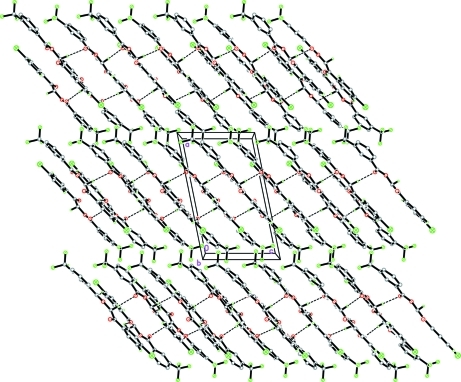
The crystal packing of the title compound, viewed along the b axis. H atoms not involved in the intermolecular interactions (dashed lines) have been omitted for clarity.

## supplementary crystallographic information

### Comment

Phenacyl benzoates derivatives are very important in identification of organic acids (Rather & Reid, 1919), they undergo photolysis in neutral and mild conditions (Sheehan & Umezaw, 1973; Ruzicka *et al.*, 2002; Litera *et al.*, 2006). They find applications in the field of synthetic chemistry for the synthesis of oxazoles, imidazoles (Huang *et al.*, 1996), benzoxazepine (Gandhi *et al.*, 1995). We hereby report the crystal structure of 2-(4-chlorophenyl)-2-oxoethyl 3-(trifluoromethyl) benzoate of potential commercial importance.

In the title compound (Fig. 1), the chlorophenyl (C1–C6/CL1) group is slightly twisted away from the benzene ring (C10–C15) with a dihedral angle of 15.50 (8)°. Bond lengths (Allen *et al.*, 1987) and angles are within the normal ranges.

In the crystal packing (Fig. 2), intermolecular C1—H1A···O1^i^, C4—H4A···O3^ii^ and C8—H8A···O3^iii^ hydrogen bonds (Table 1) link the molecules into a layer parallel to the *bc* plane.

### Experimental

A mixture of 3-(trifluoromethyl)benzoic acid (1.0 g, 0.0052 mol), potassium carbonate (0.80 g, 0.0057 mol) and 2-bromo-1-(4-chlorophenyl)ethanone (1.21 g, 0.0052 mol) in dimethylformamide (10 ml) was stirred at room temperature for 2 h. On cooling, colourless needle-shaped crystals, 2-(4-chlorophenyl)-2-oxoethyl 3-(trifluoromethyl)benzoate begin to separate. It was collected by filtration and recrystallized from ethanol. Yield: 1.60 g, 88.88%, *m.p.*: 387–388 K.

### Refinement

All the H atoms were positioned geometrically (C—H = 0.95 or 0.99 Å) and refined with a riding model, with *U*_iso_(H) = 1.2*U*_eq_(C).

### Crystal data

**Table:**

C_16_H_10_ClF_3_O_3_	*F*(000) = 696
*M**_r_* = 342.69	*D*_x_ = 1.566 Mg m^−^^3^
Monoclinic, *P*2_1_/*c*	Mo *K*α radiation, λ = 0.71073 Å
Hall symbol: -P 2ybc	Cell parameters from 3750 reflections
*a* = 14.3036 (7) Å	θ = 2.9–29.9°
*b* = 12.1335 (6) Å	µ = 0.31 mm^−^^1^
*c* = 8.5464 (4) Å	*T* = 100 K
β = 101.444 (1)°	Block, colourless
*V* = 1453.76 (12) Å^3^	0.28 × 0.16 × 0.11 mm
*Z* = 4	

### Data collection

**Table:**

Bruker SMART APEXII CCD area-detector diffractometer	3822 independent reflections
Radiation source: fine-focus sealed tube	2963 reflections with *I* > 2σ(*I*)
graphite	*R*_int_ = 0.046
φ and ω scans	θ_max_ = 29.0°, θ_min_ = 1.5°
Absorption correction: multi-scan (*SADABS*; Bruker, 2009)	*h* = −19→19
*T*_min_ = 0.919, *T*_max_ = 0.966	*k* = −10→16
15629 measured reflections	*l* = −11→11

### Refinement

**Table:**

Refinement on *F*^2^	Primary atom site location: structure-invariant direct methods
Least-squares matrix: full	Secondary atom site location: difference Fourier map
*R*[*F*^2^ > 2σ(*F*^2^)] = 0.048	Hydrogen site location: inferred from neighbouring sites
*wR*(*F*^2^) = 0.121	H-atom parameters constrained
*S* = 1.04	*w* = 1/[σ^2^(*F*_o_^2^) + (0.0486*P*)^2^ + 1.0347*P*] where *P* = (*F*_o_^2^ + 2*F*_c_^2^)/3
3822 reflections	(Δ/σ)_max_ = 0.001
208 parameters	Δρ_max_ = 0.52 e Å^−^^3^
0 restraints	Δρ_min_ = −0.33 e Å^−^^3^

### Special details

**Table:**

Experimental. The crystal was placed in the cold stream of an Oxford Cryosystems Cobra open-flow nitrogen cryostat (Cosier & Glazer, 1986) operating at 100.0 (1) K.
Geometry. All e.s.d.'s (except the e.s.d. in the dihedral angle between two l.s. planes) are estimated using the full covariance matrix. The cell e.s.d.'s are taken into account individually in the estimation of e.s.d.'s in distances, angles and torsion angles; correlations between e.s.d.'s in cell parameters are only used when they are defined by crystal symmetry. An approximate (isotropic) treatment of cell e.s.d.'s is used for estimating e.s.d.'s involving l.s. planes.
Refinement. Refinement of *F*^2^ against ALL reflections. The weighted *R*-factor *wR* and goodness of fit *S* are based on *F*^2^, conventional *R*-factors *R* are based on *F*, with *F* set to zero for negative *F*^2^. The threshold expression of *F*^2^ > σ(*F*^2^) is used only for calculating *R*-factors(gt) *etc*. and is not relevant to the choice of reflections for refinement. *R*-factors based on *F*^2^ are statistically about twice as large as those based on *F*, and *R*- factors based on ALL data will be even larger.

### Fractional atomic coordinates and isotropic or equivalent isotropic displacement parameters (Å^2^)

**Table:**

	*x*	*y*	*z*	*U*_iso_*/*U*_eq_	
Cl1	0.22643 (4)	0.86068 (4)	1.07291 (6)	0.02896 (14)	
F1	0.92991 (14)	0.87892 (13)	0.01853 (17)	0.0580 (5)	
F2	1.04777 (11)	0.85923 (17)	0.2147 (2)	0.0727 (6)	
F3	0.93614 (10)	0.73951 (11)	0.17107 (16)	0.0371 (3)	
O1	0.53911 (10)	1.07226 (11)	0.66621 (15)	0.0222 (3)	
O2	0.66428 (9)	0.93394 (11)	0.59360 (15)	0.0214 (3)	
O3	0.69311 (10)	0.76708 (11)	0.49795 (17)	0.0260 (3)	
C1	0.43295 (14)	0.83441 (15)	0.8232 (2)	0.0202 (4)	
H1A	0.4678	0.7774	0.7845	0.024*	
C2	0.36390 (14)	0.80774 (16)	0.9092 (2)	0.0225 (4)	
H2A	0.3509	0.7329	0.9297	0.027*	
C3	0.31378 (14)	0.89225 (16)	0.9650 (2)	0.0207 (4)	
C4	0.33093 (13)	1.00257 (16)	0.9356 (2)	0.0202 (4)	
H4A	0.2957	1.0593	0.9742	0.024*	
C5	0.39992 (13)	1.02787 (15)	0.8495 (2)	0.0191 (4)	
H5A	0.4122	1.1028	0.8285	0.023*	
C6	0.45214 (13)	0.94487 (15)	0.7924 (2)	0.0171 (4)	
C7	0.52797 (13)	0.97681 (15)	0.7041 (2)	0.0182 (4)	
C8	0.59082 (14)	0.88503 (15)	0.6632 (2)	0.0199 (4)	
H8A	0.6195	0.8437	0.7609	0.024*	
H8B	0.5526	0.8332	0.5868	0.024*	
C9	0.71079 (13)	0.86489 (15)	0.5125 (2)	0.0190 (4)	
C10	0.78492 (13)	0.92229 (15)	0.4433 (2)	0.0185 (4)	
C11	0.80562 (14)	1.03401 (16)	0.4691 (2)	0.0219 (4)	
H11A	0.7732	1.0758	0.5358	0.026*	
C12	0.87340 (15)	1.08395 (17)	0.3975 (3)	0.0276 (4)	
H12A	0.8872	1.1601	0.4149	0.033*	
C13	0.92112 (15)	1.02351 (17)	0.3007 (2)	0.0264 (4)	
H13A	0.9673	1.0580	0.2511	0.032*	
C14	0.90109 (14)	0.91177 (17)	0.2764 (2)	0.0228 (4)	
C15	0.83340 (13)	0.86101 (16)	0.3467 (2)	0.0198 (4)	
H15A	0.8200	0.7848	0.3292	0.024*	
C16	0.95338 (16)	0.84786 (19)	0.1713 (3)	0.0314 (5)	

### Atomic displacement parameters (Å^2^)

**Table:**

	*U*^11^	*U*^22^	*U*^33^	*U*^12^	*U*^13^	*U*^23^
Cl1	0.0281 (3)	0.0281 (3)	0.0351 (3)	−0.0017 (2)	0.0170 (2)	0.0031 (2)
F1	0.1082 (15)	0.0398 (9)	0.0358 (8)	0.0034 (9)	0.0380 (9)	−0.0024 (6)
F2	0.0272 (8)	0.0992 (15)	0.0989 (14)	−0.0158 (8)	0.0301 (8)	−0.0676 (12)
F3	0.0439 (8)	0.0256 (7)	0.0466 (8)	0.0074 (6)	0.0204 (6)	−0.0064 (6)
O1	0.0289 (7)	0.0134 (6)	0.0255 (6)	−0.0011 (5)	0.0082 (6)	0.0029 (5)
O2	0.0242 (7)	0.0165 (6)	0.0263 (6)	−0.0022 (5)	0.0121 (5)	−0.0020 (5)
O3	0.0311 (8)	0.0137 (6)	0.0362 (8)	−0.0031 (6)	0.0140 (6)	−0.0030 (6)
C1	0.0241 (9)	0.0131 (9)	0.0243 (9)	0.0023 (7)	0.0070 (7)	0.0001 (7)
C2	0.0257 (10)	0.0162 (9)	0.0272 (9)	0.0001 (7)	0.0088 (8)	0.0023 (7)
C3	0.0212 (9)	0.0214 (9)	0.0210 (8)	−0.0017 (7)	0.0078 (7)	0.0011 (7)
C4	0.0214 (9)	0.0167 (9)	0.0219 (8)	0.0035 (7)	0.0030 (7)	−0.0010 (7)
C5	0.0224 (9)	0.0140 (8)	0.0199 (8)	0.0012 (7)	0.0020 (7)	0.0013 (7)
C6	0.0200 (9)	0.0140 (8)	0.0175 (7)	−0.0005 (7)	0.0039 (6)	0.0007 (7)
C7	0.0212 (9)	0.0153 (8)	0.0173 (8)	−0.0007 (7)	0.0021 (7)	0.0010 (7)
C8	0.0232 (9)	0.0152 (9)	0.0225 (8)	−0.0007 (7)	0.0074 (7)	0.0011 (7)
C9	0.0203 (9)	0.0170 (9)	0.0200 (8)	0.0011 (7)	0.0046 (7)	−0.0008 (7)
C10	0.0186 (9)	0.0156 (9)	0.0214 (8)	0.0003 (7)	0.0038 (7)	0.0018 (7)
C11	0.0227 (9)	0.0175 (9)	0.0268 (9)	0.0015 (7)	0.0078 (7)	−0.0019 (7)
C12	0.0265 (10)	0.0168 (10)	0.0413 (11)	−0.0027 (8)	0.0112 (9)	−0.0028 (8)
C13	0.0249 (10)	0.0237 (10)	0.0323 (10)	−0.0034 (8)	0.0100 (8)	0.0020 (8)
C14	0.0210 (9)	0.0238 (10)	0.0246 (9)	0.0010 (8)	0.0068 (7)	−0.0039 (8)
C15	0.0216 (9)	0.0161 (9)	0.0219 (8)	0.0000 (7)	0.0043 (7)	−0.0014 (7)
C16	0.0298 (11)	0.0325 (12)	0.0356 (11)	−0.0061 (9)	0.0155 (9)	−0.0119 (9)

### Geometric parameters (Å, °)

**Table:**

Cl1—C3	1.7364 (19)	C5—H5A	0.9500
F1—C16	1.336 (3)	C6—C7	1.490 (3)
F2—C16	1.335 (3)	C7—C8	1.515 (3)
F3—C16	1.338 (3)	C8—H8A	0.9900
O1—C7	1.222 (2)	C8—H8B	0.9900
O2—C9	1.344 (2)	C9—C10	1.486 (3)
O2—C8	1.435 (2)	C10—C15	1.394 (3)
O3—C9	1.215 (2)	C10—C11	1.396 (3)
C1—C2	1.381 (3)	C11—C12	1.385 (3)
C1—C6	1.403 (3)	C11—H11A	0.9500
C1—H1A	0.9500	C12—C13	1.383 (3)
C2—C3	1.389 (3)	C12—H12A	0.9500
C2—H2A	0.9500	C13—C14	1.393 (3)
C3—C4	1.393 (3)	C13—H13A	0.9500
C4—C5	1.378 (3)	C14—C15	1.381 (3)
C4—H4A	0.9500	C14—C16	1.495 (3)
C5—C6	1.398 (3)	C15—H15A	0.9500
			
C9—O2—C8	115.72 (14)	O3—C9—O2	123.20 (17)
C2—C1—C6	120.70 (17)	O3—C9—C10	124.57 (17)
C2—C1—H1A	119.6	O2—C9—C10	112.22 (16)
C6—C1—H1A	119.6	C15—C10—C11	119.79 (17)
C1—C2—C3	118.83 (18)	C15—C10—C9	117.65 (17)
C1—C2—H2A	120.6	C11—C10—C9	122.55 (17)
C3—C2—H2A	120.6	C12—C11—C10	120.03 (18)
C2—C3—C4	121.75 (18)	C12—C11—H11A	120.0
C2—C3—Cl1	119.64 (15)	C10—C11—H11A	120.0
C4—C3—Cl1	118.61 (15)	C13—C12—C11	120.28 (19)
C5—C4—C3	118.75 (17)	C13—C12—H12A	119.9
C5—C4—H4A	120.6	C11—C12—H12A	119.9
C3—C4—H4A	120.6	C12—C13—C14	119.61 (19)
C4—C5—C6	120.98 (17)	C12—C13—H13A	120.2
C4—C5—H5A	119.5	C14—C13—H13A	120.2
C6—C5—H5A	119.5	C15—C14—C13	120.71 (18)
C5—C6—C1	118.99 (17)	C15—C14—C16	120.48 (19)
C5—C6—C7	118.83 (16)	C13—C14—C16	118.80 (18)
C1—C6—C7	122.16 (16)	C14—C15—C10	119.58 (18)
O1—C7—C6	121.81 (17)	C14—C15—H15A	120.2
O1—C7—C8	121.41 (17)	C10—C15—H15A	120.2
C6—C7—C8	116.78 (15)	F2—C16—F1	106.7 (2)
O2—C8—C7	107.97 (14)	F2—C16—F3	106.1 (2)
O2—C8—H8A	110.1	F1—C16—F3	105.40 (17)
C7—C8—H8A	110.1	F2—C16—C14	112.24 (17)
O2—C8—H8B	110.1	F1—C16—C14	112.5 (2)
C7—C8—H8B	110.1	F3—C16—C14	113.35 (18)
H8A—C8—H8B	108.4		
			
C6—C1—C2—C3	−0.1 (3)	O2—C9—C10—C15	−175.47 (15)
C1—C2—C3—C4	0.5 (3)	O3—C9—C10—C11	−177.10 (18)
C1—C2—C3—Cl1	179.84 (15)	O2—C9—C10—C11	3.2 (2)
C2—C3—C4—C5	−0.4 (3)	C15—C10—C11—C12	0.7 (3)
Cl1—C3—C4—C5	−179.73 (14)	C9—C10—C11—C12	−178.00 (18)
C3—C4—C5—C6	−0.1 (3)	C10—C11—C12—C13	−0.2 (3)
C4—C5—C6—C1	0.4 (3)	C11—C12—C13—C14	−0.4 (3)
C4—C5—C6—C7	−178.17 (16)	C12—C13—C14—C15	0.7 (3)
C2—C1—C6—C5	−0.3 (3)	C12—C13—C14—C16	179.9 (2)
C2—C1—C6—C7	178.24 (17)	C13—C14—C15—C10	−0.2 (3)
C5—C6—C7—O1	−7.6 (2)	C16—C14—C15—C10	−179.49 (17)
C1—C6—C7—O1	173.85 (17)	C11—C10—C15—C14	−0.4 (3)
C5—C6—C7—C8	172.43 (16)	C9—C10—C15—C14	178.31 (16)
C1—C6—C7—C8	−6.1 (2)	C15—C14—C16—F2	−128.0 (2)
C9—O2—C8—C7	−163.11 (14)	C13—C14—C16—F2	52.7 (3)
O1—C7—C8—O2	5.7 (2)	C15—C14—C16—F1	111.7 (2)
C6—C7—C8—O2	−174.34 (14)	C13—C14—C16—F1	−67.6 (2)
C8—O2—C9—O3	−0.2 (3)	C15—C14—C16—F3	−7.8 (3)
C8—O2—C9—C10	179.50 (14)	C13—C14—C16—F3	172.98 (18)
O3—C9—C10—C15	4.2 (3)		

### Hydrogen-bond geometry (Å, °)

**Table:**

*D*—H···*A*	*D*—H	H···*A*	*D*···*A*	*D*—H···*A*
C1—H1A···O1^i^	0.95	2.53	3.205 (2)	128.
C4—H4A···O3^ii^	0.95	2.53	3.289 (2)	137.
C8—H8A···O3^iii^	0.99	2.48	3.474 (2)	177.

Symmetry codes: (i) −*x*+1, *y*−1/2, −*z*+3/2; (ii) −*x*+1, *y*+1/2, −*z*+3/2; (iii) *x*, −*y*+3/2, *z*+1/2.
